# A Retrospective Analysis of 581 Cases of Esophagogastric Variceal Bleeding Complicated by Portal Vein Thrombosis at a Single Center

**DOI:** 10.1155/cjgh/8185878

**Published:** 2026-08-23

**Authors:** Jiayi Zhang, Yingying He, Shanshan Wu, Derun Kong

**Affiliations:** ^1^ Department of Gastroenterology, The First Affiliated Hospital of Anhui Medical University, Hefei 230001, China, ahmu.edu.cn

**Keywords:** esophagogastric variceal bleeding, liver cirrhosis, nomogram, portal vein thrombosis

## Abstract

**BACKGROUND:**

Esophagogastric variceal bleeding (EGVB) and portal vein thrombosis (PVT) are common complications of liver cirrhosis and frequently coexist, with reported prevalence rate up to 32.5%. Their coexistence complicates clinical management and is associated with adverse outcomes. Early identification of PVT may improve patient management and prognosis.

**OBJECTIVE:**

To investigate the prevalence, clinical characteristics, and risk factors of PVT in patients with EGVB and to develop a nomogram for the early identification of PVT.

**METHODS:**

This retrospective case–control study included 581 consecutive cirrhotic patients with EGVB treated at a single center. Patients were classified into PVT and non‐PVT groups according to imaging findings. The demographic characteristics, clinical manifestations, laboratory parameters, and treatment histories were compared between the two groups. Risk factors associated with PVT were identified using logistic regression analysis, and a predictive nomogram was subsequently developed to estimate the probability of PVT occurrence.

**RESULTS:**

Among the 581 patients, 201 patients (34.6%) had concomitant PVT. The median age was 55 years (interquartile range, 48.0–64.0 years), and 384 (66.1%) were male. Compared with the non‐PVT group, patients with PVT more frequently presented with abdominal pain (15.9% vs. 10.3%, *p* = 0.048) and hepatic hydrothorax (18.9% vs. 12.4%, *p* = 0.034) than the non‐PVT group. Multivariable analysis identified previous splenectomy (OR = 4.615, 95% CI: 2.418–8.806; *p* < 0.001) and endoscopic treatment (OR = 2.414; 95% CI 1.644–3.545; *p* < 0.001) were as independent risk factors for PVT. Patients with Yerdel Grade IV had a significantly higher prevalence of splenectomy than those with Yerdel Grade I (59.1% vs. 23.4%). Similarly, the prevalence of PVT was significantly higher among patients with a history of endoscopic treatment than among those without (46.9% vs. 26.6%, *p* < 0.001). D‐dimer and fibrin degradation product (FDP) levels were significantly elevated in the PVT group. Receiver operating characteristic analysis identified optimal cutoff values of 0.995 μg/mL for D‐dimer and 2.665 μg/mL for FDP. A nomogram incorporating significant predictors demonstrated moderate discrimination for identifying PVT (AUC = 0.751).

**CONCLUSION:**

PVT was present in approximately one‐third of cirrhotic with EGVB. Previous splenectomy and endoscopic intervention were independently associated with PVT, while abdominal pain and hepatic hydrothorax were more common clinical manifestations. The nomogram showed moderate diagnostic performance and may facilitate early identification of PVT; however, external validation is required before routine clinical application.

## 1. Introduction

Esophagogastric variceal bleeding (EGVB) and portal vein thrombosis (PVT) are two major complications of liver cirrhosis. Despite substantial advances in the prevention and management of EGVB, it remains a life‐threatening event with a reported mortality of 25%–50% among cirrhotic patients [1]. PVT, defined as thrombosis within the extrahepatic portal venous system, ranges from asymptomatic partial obstruction to complete obstruction. It may aggravate portal hypertension, precipitate hepatic decompensation, increase the risk of recurrent variceal bleeding, and lead to intestinal ischemia. The prevalence of PVT varies widely among cirrhotic patients, influenced by patient characteristics and diagnostic methods, with reported rates spanning from 1% to 26% across different studies [2–4]. Our prior research has revealed that it coexists with EGVB in approximately 24.1%–32.5% of cirrhotic patients [5], suggesting that these complications frequently occur together and may mutually exacerbate disease progression. Early detection and prompt treatment of PVT can mitigate the progression of portal hypertension and prevent severe complications such as mesenteric ischemia and intestinal infarction [6, 7]. Although anticoagulation has been demonstrated to be safe and effective in carefully selected cirrhotic patients with PVT after bleeding control [8–11], its use remains limited in patients with EGVB because of concerns regarding recurrent hemorrhage. Consequently, the coexistence of EGVB and PVT presents substantial diagnostic and therapeutic challenges, especially for patients with active bleeding. Nevertheless, studies specifically investigating cirrhotic patients with concomitant EGVB and PVT remain scarce, and the prevalence, clinical characteristics, and risk factors of PVT in this high‐risk population have not been comprehensively characterized.

Several factors have been reported to contribute to development of PVT in cirrhosis, including reduced portal vein flow velocity < 15 cm/s [12], history of EGVB [12], prior splenectomy [13, 14], elevated D‐dimer (DD) levels [15], *β*‐blocker usage [16], endoscopic variceal ligation (EVL) [13], and enlarged splenic vein diameters [13]. However, whether these factors remain predictive in patients with EGVB has not been adequately investigated, particularly with respect to prior endoscopic treatment. Therefore, the present study retrospectively analyzed a large cohort of cirrhotic patients with EGVB to determine the prevalence, clinical characteristics, and risk factors of concomitant PVT. Furthermore, we developed a predictive nomogram to facilitate the early identification of PVT in this high‐risk population.

## 2. Materials and Methods

### 2.1. Study Population

This retrospective study enrolled consecutive patients diagnosed with cirrhosis, who presented with EGVB and underwent endoscopic treatment at the First Affiliated Hospital of Anhui Medical University between January 2018 and December 2022. The study was conducted in accordance with the Declaration of Helsinki (seventh revision) and obtained approval from the Ethics Committee of the First Affiliated Hospital of Anhui Medical University (Approval No. PJ2024‐09‐53).

Inclusion criteria defined as follows: (1) age 18–80 years; patients younger than 18 years were excluded as minors, and those older than 80 years were excluded because elderly patients in this age group are more likely to present with multiple severe comorbidities. To improve sample homogeneity and the interpretability of the results, the age range was restricted to 18–80 years; (2) diagnosed cirrhosis complicated by EGVB, verified endoscopically in accordance with established diagnostic criteria [17]; and (3) comprehensive clinical data available for analysis.

Exclusion criteria were defined as follows: (1) cirrhosis secondary to venous obstruction or thrombosis‐related disease, such as Budd–Chiari syndrome; (2) poor overall health, including uncontrolled active bleeding, multiple‐organ failure, hepatic encephalopathy (Grades III and IV), and malignancies such as hepatocellular carcinoma; (3) history of transjugular intrahepatic portosystemic shunt (TIPS) procedure or liver transplantation; and (4) extrahepatic diseases associated with PVT, such as myeloproliferative neoplasms and thrombophilia [18–21].

PVT was diagnosed by color Doppler ultrasonography, computed tomography angiography (CTA), or magnetic resonance angiography (MRA). Each patient underwent at least one of these imaging examinations. All imaging findings were independently reviewed by experienced radiologists and the diagnosis and classification of PVT were established. Based on the occurrence or absence of concomitant PVT during hospitalization, patients were divided into the PVT group and the non‐PVT group.

### 2.2. Data Sources and Collection Methodology

Comprehensive data were retrospectively extracted from the electronic medical records of all enrolled patients using a standardized data collection form. The collected variables included demographic characteristics, medical history, clinical manifestations, laboratory parameters, imaging and endoscopic findings, therapeutic interventions, and treatment outcomes.

Demographic characteristics specifically included age and sex. Medical history information was meticulously documented, highlighting the etiology of cirrhosis, previous splenectomy, and previous endoscopic treatment modalities (particularly noting whether endoscopic treatment was adopted, as well as the specific measures of the most recent procedure). Viral etiology was subtyped as hepatitis B virus (HBV)–related or hepatitis C virus (HCV)–related based on serology and/or nucleic acid testing. Liver function was assessed using the Child–Pugh classification. Clinical manifestations at admission were recorded, including abdominal pain, fever, ascites, and hepatic hydrothorax. Laboratory parameters obtained at admission included DD, international normalized ratio, fibrin degradation product (FDP), prothrombin time, activated partial thromboplastin time, thromboplastin time, antithrombin III, white blood cell count (WBC), platelet count (PLT), albumin, total bilirubin, alkaline phosphatase, and other pertinent parameters related to coagulation function and hepatic status.

Radiological examinations, endoscopic findings, and treatment records were reviewed to determine the presence and severity of PVT, characteristics of variceal bleeding, and treatment history. All data were independently reviewed by two investigators, and any discrepancies were resolved by consensus.

### 2.3. Definitions

The extent of PVT was classified according to the Yerdel grading system, which is as follows:

1. Yerdel Grade I: Minimal or partial thrombosis involving less than 50% of the lumen of the thrombosed vessel, with or without minor extension into the superior mesenteric vein (SMV).
2. Yerdel Grade II: Greater than 50% obstruction of the portal vein, encompassing complete thrombosis of the portal vein, with or without minor extension into the SMV.
3. Yerdel Grade III: Complete thrombosis of both the portal vein and the proximal segment of the SMV, with unobstructed distal segment of the SMV.
4. Yerdel Grade IV: Complete thrombosis of both the portal vein and the entire SMV [4, 20, 22].

### 2.4. Statistical Analysis

Statistical analyses were performed using IBM SPSS Statistics Version 27.0 (IBM, Armonk, NY, USA) and R Version 4.3.3 (R Foundation for Statistical Computing, Vienna, Austria). A two‐sided *p* value < 0.05 was considered statistically significant. Based on the normality, normally and nonnormally distributed quantitative data were described in mean ± standard deviation ($\overset{¯}{x}\pm SD$) and median (interquartile range [IQR]). Comparisons were made using the independent‐samples *t*‐test or Mann–Whitney *U* test, as appropriate. Categorical variables are expressed as frequencies and percentages and were compared using or chi‐square or Fisher’s exact test.

Potential risk factors associated with concomitant PVT were first evaluated using univariable logistic regression. Variables with *p* < 0.05 in the univariable analysis, together with clinically relevant variables, were entered into a multivariable logistic regression model to identify independent predictors of PVT. Odds ratios (ORs) with corresponding 95% confidence intervals (CIs) were calculated. A predictive nomogram was constructed based on the independent predictors identified in the multivariable logistic regression model. Model discrimination was evaluated using the area under the receiver operating characteristic (ROC) curve (AUC), while calibration was assessed using calibration plots with bootstrap internal validation based on 1000 resamples. Calibration performance was further quantified using the corrected slope and the Brier score.

## 3. Results

### 3.1. Baseline Characteristics

**FIGURE 1:**
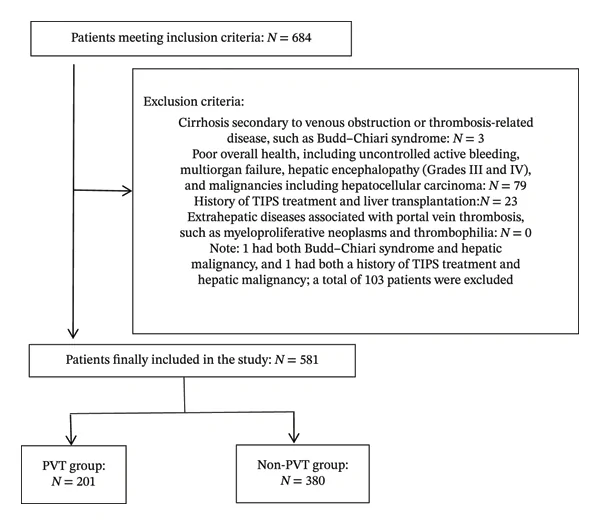
Flowchart for the inclusion of eligible population in this study.

A total of 684 patients with cirrhotic EGVB were initially screened between January 2018 and December 2022. After applying the predefined exclusion criteria, 581 eligible patients were included in the final analysis (Figure 1). The baseline characteristics of the study population are summarized in Table 1. The median age was 55 years (IQR, 48.0–64.0 years), and 384 patients (66.1%) were male. Furthermore, 228 patients (39.2%) had undergone previous endoscopic treatment for varices. Among these, 98 patients (43.0%) had undergone EVL, while 130 patients (57.0%) had received endoscopic injection sclerotherapy (EIS). Notably, 201 patients (34.6%) with concomitant PVT were allocated to the PVT group, while the remaining 380 patients (65.4%) comprised the non‐PVT group.

**TABLE 1:** Clinical characteristics of eligible cirrhotic EGVB patients.

Diagnostic trait	Patients (*N* = 581)
Males (*n*, %)	384 (66.1)
Age, years (median [interquartile range])	55 (48.0–64.0)
Causes of cirrhosis (*n*, %)	
Viral hepatitis	319 (54.9)
Hepatitis B virus	309 (53.2)
Hepatitis C virus	10 (1.7)
Nonviral hepatitis	262 (45.1)
Child–Pugh score (median [interquartile range])	7 (6.0–8.0)
Child–Pugh classification A/B/C (*n*, %)	178 (30.6)/346 (59.6)/57 (9.8)
PVT (*n*, %)	201 (34.6)
Yerdel Grade I	64 (31.8)
Yerdel Grade II	79 (39.3)
Yerdel Grade III	36 (17.9)
Yerdel Grade IV	22 (10.9)
Splenectomy (*n*, %)	89 (15.3)
Previous endoscopic treatment (*n*, %)	228 (39.2)
Last endoscopic treatment modality (*n*, %)	
EVL	98 (43.0)
EIS	130 (57.0)

Abbreviations: EIS, endoscopic injection sclerotherapy; EVL, endoscopic variceal ligation.

### 3.2. Prevalence of PVT According to Endoscopic Treatment History

This study further investigated the impact of endoscopic treatment on the prevalence of concomitant PVT in EGVB cases (Table 2). Among patients with endoscopic treatment history for EGVB, 107 of 228 (46.9%) had PVT, compared with 94 of 353 (26.6%) patients without previous endoscopic treatment (*p* < 0.001). When stratified by treatment modality, 48 patients (49.0%) who underwent EVL and 59 patients (45.4%) who underwent EIS had concomitant PVT. Notably, patients without endoscopic treatment exhibited a lower prevalence of PVT than those with EVL (49.0% vs. 26.6%, *p* < 0.010) and EIS (45.4% vs. 26.6%, *p* < 0.010), indicating a statistically significant difference.

**TABLE 2:** Effect of endoscopic treatment on the prevalence of PVT in cirrhotic EGVB patients.

		PVT group	Non‐PVT group	Chi‐square value	*p* *value*
Yes vs. no	Yes	107 (46.9%)	121 (53.1%)	25.231	< 0.001
	No	94 (26.6%)	259 (73.4%)		

EVL vs. EIS	EVL	48 (49.0%)	50 (51.0%)	0.290	0.590
	EIS	59 (45.4%)	71 (54.6%)		

EVL vs. no	EVL	48 (49.0%)	50 (51.0%)	17.763	< 0.001
	No	94 (26.6%)	259 (73.4%)		

EIS vs. no	EIS	59 (45.4%)	71 (54.6%)	15.443	< 0.001
	No	94 (26.6%)	259 (73.4%)		

Abbreviations: EIS, endoscopic injection sclerotherapy; EVL, endoscopic variceal ligation.

### 3.3. Clinical Characteristics of Patients With EGVB and Concomitant PVT

Clinical characteristics of patients were compared between the PVT and non‐PVT groups (Table 3). Specifically, abdominal pain (32 [15.9%] vs. 39 [10.3%], *P* = 0.048), splenectomy (67 [33.3%] vs. 22 [5.8%], *p* < 0.001), and hepatic hydrothorax (38 [18.9%] vs. 47 [12.4%], *p* = 0.034) were more frequently observed in patients with PVT compared to those without PVT.

**TABLE 3:** Characteristics of clinical manifestations in cirrhotic EGVB patients in the PVT and non‐PVT groups.

Symptom or sign	PVT group	Non‐PVT group	Total	*p* *value*
Abdominal pain	32 (15.9%)	39 (10.3%)	71 (12.2%)	0.048
Fever	8 (4.0%)	5 (1.3%)	13 (2.2%)	0.077
Ascites	116 (57.7%)	222 (58.4%)	338 (58.2%)	0.869
Splenectomy	67 (33.3%)	22 (5.8%)	89 (15.3%)	< 0.001
Hepatic hydrothorax	38 (18.9%)	47 (12.4%)	85 (14.6%)	0.034

This study also examined the clinical characteristics of patients with cirrhotic EGVB complicated by PVT across different Yerdel grades (Table 4). Previous splenectomy was significantly more common in patients with Yerdel Grade IV PVT =  compared to those with Yerdel Grade I PVT (59.1% vs. 23.4%, *p* = 0.002).

**TABLE 4:** Characteristics of clinical manifestations in patients with cirrhotic EGVB complicated by PVT with different Yerdel classifications.

	Yerdel Grade I	Yerdel Grade II	Yerdel Grade III	Yerdel Grade IV	*p* *value*
Abdominal pain	12 (18.8%)	8 (10.1%)	7 (19.4%)	5 (22.7%)	0.268
Fever	1 (1.6%)	4 (5.1%)	3 (8.3%)	0 (0.0%)	0.270
Ascites	28 (43.8%)	51 (64.6%)	24 (66.7%)	13 (59.1%)	0.050
Splenectomy	15 (23.4%)	25 (31.6%)	14 (38.9%)	13 (59.1%)	0.019^∗^
Hepatic hydrothorax	13 (20.3%)	17 (21.5%)	4 (11.1%)	4 (18.2%)	0.608

*Note:* A significant difference for splenectomy was observed between Yerdel Grade I and Yerdel Grade IV (adjusted *p* < 0.05).

^∗^Pairwise comparisons were performed using Bonferroni correction.

### 3.4. Laboratory Characteristics and Diagnostic Performance

**FIGURE 2:**
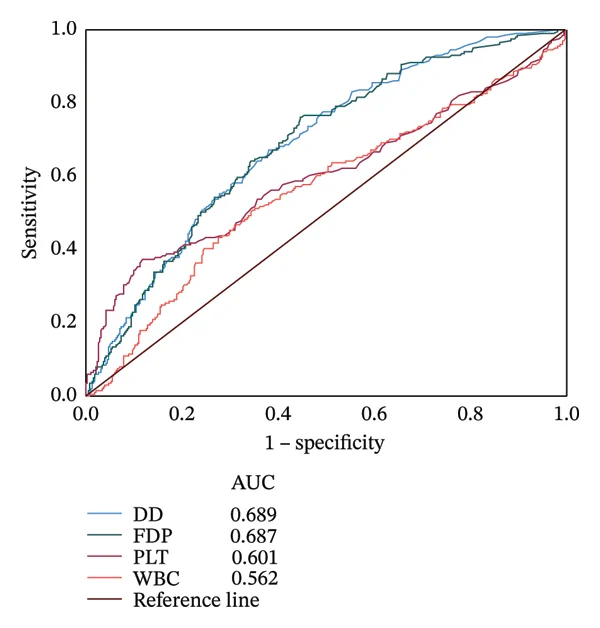
Predictive value of DD, FDP, PLT, and WBC for PVT. *Note:* PLT, platelet count; WBC, white blood cell count. Abbreviations: DD, D‐dimer; FDP, fibrin degradation product; PVT, portal vein thrombosis.

As depicted in Table 5, the PVT group exhibited significantly elevated levels of DD (1.69 vs. 0.68 μg/mL, *p* < 0.001), FDP (5.52 vs. 2.43 μg/mL, *p* < 0.001), PLT (81.00 [46.00–179.00] vs. 65.00 [45.00–92.75] × 10^9^/L, *p* < 0.001), and WBC (3.71 [2.18–5.55] vs. 3.06 [2.03–4.43] × 10^9^/L *p* = 0.014] compared to the non‐PVT group. ROC curve analysis was performed to evaluate the diagnostic performance of these laboratory parameters (Figure 2). DD demonstrated the highest discriminative ability for identifying PVT (AUC = 0.689), followed by FDP (AUC = 0.687), PLT (AUC = 0.601), and WBC (AUC = 0.562). However, the AUC values were all below 0.7, indicating only modest discriminative ability. The optimal cutoff values determined by the Youden index are shown in Table 6. A DD level that exceeded 0.995 μg/mL predicted concomitant PVT, with a sensitivity of 67.2% and a specificity of 61.8%. Similarly, PVT complication might be anticipated when FDP levels surpassed 2.665 μg/mL, exhibiting a sensitivity of 76.1% and a specificity of 55.3%. Additionally, PLT above 135.5 × 10^9^/L predicted PVT with a sensitivity of 50.7% and a specificity of 65.3%.

**TABLE 5:** Comparison of laboratory indices in patients with cirrhotic EGVB combined with PVT and patients without PVT.

Laboratory indicators	PVT group	Non‐PVT group	*Z*	*p* *value*
PT, *s*	14.90 (13.55–16.25)	14.80 (13.80–16.10)	−0.164	0.870
APTT, %	68.10 (59.20–76.70)	68.10 (60.0–76.30)	−0.530	0.596
INR	1.19 (1.07–1.31)	1.19 (1.09–1.29)	−0.409	0.683
APTT, s	30.70 (27.90–36.50)	31.80 (28.10–38.40)	−1.653	0.098
FIB, g/mL	1.77 (1.45–2.27)	1.74 (1.44–2.19)	−0.767	0.443
TT, *s*	19.20 (17.20–20.80)	19.30 (17.90–20.60)	−0.694	0.488
AT‐III, %	61.30 (51.55–71.10)	62.83 (54.13–74.34)	−1.482	0.138
DD, μg/mL	1.69 (0.73–3.41)	0.68 (0.33–1.61)	−7.491	< 0.001
FDP, μg/mL	5.52 (2.70–10.82)	2.43 (1.37–5.31)	−7.419	< 0.001
PLT, × 10^9^/L	81.00 (46.00–179.00)	65.00 (45.00–92.75)	−4.026	< 0.001
Hb, g/L	82.0 (68.0–100.0)	81 (67.0–99.8)	−0.237	0.812
WBC, × 10^9^/L	3.71 (2.18–5.55)	3.06 (2.03–4.43)	−2.469	0.014
ALB, g/L	33.20 (29.60–36.35)	34.00 (30.30–37.40)	−1.605	0.108
TBil, μmol/L	18.67 (13.22–26.4)	18.73 (13.79–27.90)	−0.932	0.351
ALP, U/L	95.00 (68.50–136.00)	93.00 (72.00–128.00)	−0.070	0.944

*Note:* EGVB, esophagogastric variceal bleeding; ALB, albumin; ALP, alkaline phosphatase; AT‐III, antithrombin III; FIB, fibrinogen; Hb, hemoglobin; PLT, platelet count; Tbil, total bilirubin; WBC, white blood cell count.

Abbreviations: APTT, activated partial thromboplastin time; DD, D‐dimer; FDP, fibrin degradation product; INR, international normalized ratio; PT, prothrombin time; PVT, portal vein thrombosis; TT, thrombin time.

**TABLE 6:** Predictive value of laboratory parameters for PVT.

Laboratory indicators	AUC	*p* *value*	Threshold value	Sensitivity	Specificity
	(95% CI)		(Cutoff value)	(%)	(%)
DD, μg/mL	0.689	< 0.001	0.995	67.2	61.8
FDP, μg/mL	0.687	< 0.001	2.665	76.1	55.3
PLT, × 10^9^/L	0.601	< 0.001	135.5	37.3	88.2
WBC, × 10^9^/L	0.562	0.014	3.68	50.7	65.3

*Note:* PLT, platelet count; WBC, white blood cell count.

Abbreviations: DD, D‐dimer; FDP, fibrin degradation product; PVT, portal vein thrombosis.

### 3.5. Analysis of Factors Influencing EGVB‐Associated PVT and Nomogram Prediction Model

Table 7 presents the analysis of risk factors associated with PVT in patients with EGVB. In univariate analysis, age (OR = 0.983; 95% CI: 0.969–0.997, *p* = 0.017), viral hepatitis‐related cirrhosis (OR = 1.435; 95% CI: 1.014–2.030, *p* = 0.042), previous splenectomy (OR = 8.136; 95% CI 4.833–13.698, *p* < 0.001), previous endoscopic treatment (OR = 2.437; 95% CI 1.715–3.462, *p* < 0.001), elevated DD level (OR = 1.141; 95% CI 1.068–1.218. *p* < 0.001), increased FDP level (OR = 1.045; 95% CI 1.023–1.068, *p* < 0.001), and higher PLT (OR = 1.007; 95% CI 1.004–1.009, *p* < 0.001) were associated with PVT in EGVB patients. In the multivariate analysis, previous splenectomy (OR = 4.615; 95% CI 2.418–8.086, *p* < 0.001) and endoscopic treatment (OR = 2.414; 95% CI 1.644–3.545, *p* < 0.001) remained independent predictors of concomitant PVT.

**TABLE 7:** Analysis of factors associated with EGVB complicating PVT.

Diagnostic trait	Univariate analysis			Multifactorial analysis		
	OR	95% CI	*p* *value*	OR	95% CI	*p* *value*
Sex (male/female)	1.325	0.917–1.913	0.134			
Age (years)	0.983	0.969–0.997	0.017	1.002	0.986–0.971	0.090
Etiology of cirrhosis (viral hepatitis vs. nonviral hepatitis)	1.435	1.014–2.030	0.042	1.359	0.916–2.018	0.128
Splenectomy	8.136	4.833–13.698	< 0.001	4.615	2.418–8.806	< 0.001
Previous endoscopic treatment	2.437	1.715–3.462	< 0.001	2.414	1.644–3.545	< 0.001
DD, μg/mL	1.141	1.068–1.218	< 0.001	1.090	0.946–1.257	0.233
FDP, μg/mL	1.045	1.023–1.068	< 0.001	1.008	0.962–1.056	0.728
PLT, × 10^9^/L	1.007	1.004–1.009	< 0.001	1.002	1.000–1.005	0.069
WBC, × 10^9^/L	0.998	0.981–1.015	0.784			
Child–Pugh score (median [IQR])	1.012	0.906–1.130	0.833			
Child–Pugh Grades A/B/C			0.690			
Child–Pugh B vs. Child–Pugh A	1.113	0.758–1.633	0.585			
Child–Pugh C vs. Child–Pugh A	1.300	0.701–2.414	0.405			

*Note:* PLT, platelet count; WBC, white blood cell count.

Abbreviations: DD, D‐dimer; FDP, fibrin degradation product; PVT, portal vein thrombosis.

**FIGURE 3:**
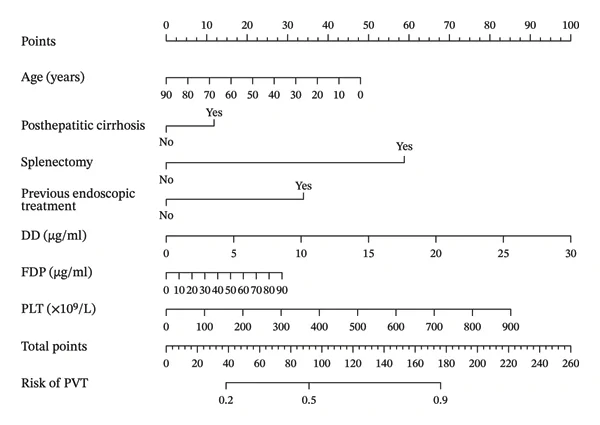
A column‐line graphical model designed to predict PVT occurrence. Note: PLT, platelet count; WBC, white blood cell count. Abbreviations: DD, D‐dimer; FDP, fibrin degradation product; PVT, portal vein thrombosis.

**FIGURE 4:**
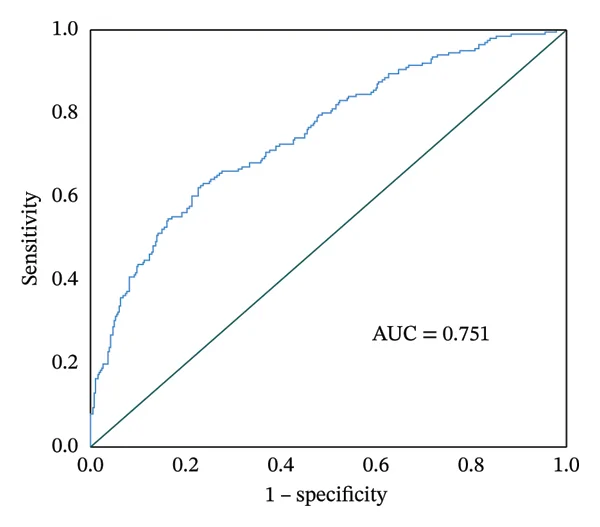
ROC curve of the nomogram prediction model for PVT, with an AUC of 0.751 (95% CI 0.709–0.793).

**FIGURE 5:**
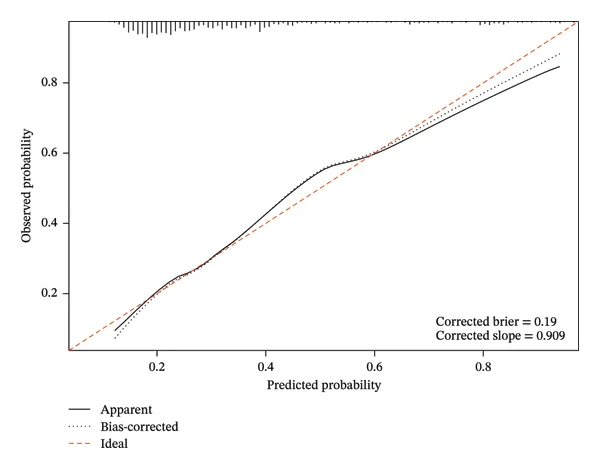
Calibration curve of the prediction model based on bootstrap internal validation (*B* = 1000).

Based on the results of univariate analysis, a nomogram was established to predict the probability of PVT (Figure 3). The performance of this model was evaluated using a ROC curve (Figure 4), yielding an AUC of 0.751 (95% CI: 0.709–0.793), indicating its moderate discriminative ability in predicting PVT in EGVB patients. Internal validation using 1000 bootstrap resamples showed good agreement between the predicted and observed probabilities (Figure 5). The optimism‐corrected calibration slope was 0.909, indicating minimal overfitting, and the corrected Brier score was 0.190, suggesting acceptable overall predictive accuracy.

## 4. Discussion

EGVB and PVT represent a frequent complication in patients with cirrhosis and constitute a primary contributor to the heightened mortality rates observed in this patient population. However, most existing studies have focused on cirrhosis complicated by PVT alone, while evidence regarding patients with concomitant EGVB and PVT remains limited. In this context, we conducted a large single‐center study including 581 cirrhotic patients with EGVB to investigate the prevalence, clinical characteristics, and risk factors of PVT and to develop a predictive model for early identification.

This study found a relatively high incidence of PVT in patients with liver cirrhosis and EGVB (34.6%). Compared with non‐PVT patients, those with PVT more often had a history of splenectomy and previous endoscopic treatment, and more frequently presented with abdominal pain and hepatic hydrothorax, along with higher DD and FDP levels. A nomogram with moderate predictive performance was developed, incorporating age, posthepatitic cirrhosis, splenectomy, previous endoscopic treatment, DD, FDP, and PLT. These findings may support early identification of PVT in cirrhosis and EGVB, enabling timely intervention and potentially improving outcomes. The incidence of PVT among the enrolled 581 EGVB patients was 34.6%, exceeding one‐third of the cohort, which is higher than that reported previously. Turon et al. [12] reported the incidence of 1.6%, 6%, and 8.3% at 1, 3, and 5 years, respectively, 72% of which were classified as Child–Pugh Grade A. Similarly, Noronha et al. [23] reported 3.7% at 1 year and 7.6% at 3 years in the incidence, 76.3% of which showed Child–Pugh Class A. In contrast, more than 60% of patients in our cohort were classified as Child–Pugh Class B or C, suggesting more advanced liver dysfunction. The higher prevalence of PVT observed in our study may therefore be attributed to more severe portal hypertension and poorer hepatic reserve in patients presenting with EGVB.

Furthermore, among the 201 patients with PVT, 15.9% (32 patients) experienced abdominal pain and 18.9% (38 patients) developed hepatic hydrothorax, which were higher compared to those without PVT. These findings suggest that EGVB accompanied by abdominal pain and hepatic hydrothorax should prompt a thorough evaluation for the presence of PVT. Consistently, Amitrano et al. [24] reported that 18% of cirrhotic patients with PVT presented with acute abdominal pain, supporting the clinical relevance of this symptom.

Importantly, previous splenectomy and endoscopic treatment were identified as independent risk factors for PVT. In our cohort, the prevalence of PVT following splenectomy was 33.3%, and a higher proportion of patients with Yerdel Grade IV PVT had a history of splenectomy compared with those with Yerdel Grade I (59.1% vs. 23.4%). These findings suggest a potential association between splenectomy and more extensive thrombotic involvement. This observation is consistent with current guidelines and previous studies reporting that splenectomy is associated with an increased risk of portal venous system thrombosis and more extensive thrombus formation [25–28]. The underlying mechanisms may involve postoperative hypercoagulability, platelet activation, endothelial dysfunction, and altered portal hemodynamics following splenectomy [29].

Additionally, this study revealed a significantly higher prevalence of PVT in patients with a history of endoscopic treatment for EGVB compared to those without (46.9% vs. 26.6%). No significant difference was observed between EVL and EIS (49.0% vs. 45.4%). Some studies have suggested that both EVL and EIS are associated with an increased risk of PVT in cirrhosis [13, 30, 31]. Potential mechanisms linking endoscopic therapy to PVT include alterations in portal hemodynamics, endothelial injury, and possible embolic migration [30, 32, 33]. Previous studies have reported inconsistent findings regarding the risk of PVT following different endoscopic therapies. A meta‐analysis [34] suggested that the incidence of PVST after EIS was higher than that after EVL (11.7% vs. 5.5%). In contrast, a retrospective case–control study [30] found that patients with PVST had a higher proportion of prior EVL alone or EVL combined with endoscopic cyanoacrylate injection. Overall, the comparative impact of different endoscopic treatment modalities on PVT risk remains inconclusive and warrants further investigation.

DD and FDP were significantly associated with the presence of PVT in patients with cirrhotic EGVB. Patients in the PVT group exhibited significantly elevated DD levels compared to those in the non‐PVT group (1.69 vs. 0.68 μg/mL), corresponding to approximately threefold higher than the upper limit of normal (< 0.5 μg/mL). ROC analysis revealed an optimal threshold value of DD for predicting PVT of 0.995 μg/mL, suggesting its potential utility as a sensitive diagnostic marker for PVT. Comparison with previous studies by Malaguarnera et al. [35] and Zhang et al. [36], our findings further refine the diagnostic threshold of DD in this specific high‐risk population. Simultaneously, PVT patients demonstrated higher FDP level than those without PVT (5.52 vs. 2.43 μg/mL), although FDP levels remained within the normal range (0–5 μg/mL). The optimal cutoff value of FDP was 2.665 μg/mL for predicting PVT, indicating that even subtle elevations within the normal range may reflect an activated coagulation–fibrinolysis balance in cirrhotic patients with portal thrombosis.

A nomogram predictive model incorporating age, posthepatitic cirrhosis, splenectomy, previous endoscopic treatment, DD, FDP, and PLT, with an AUC of 0.751 suggests that combining clinical and laboratory parameters may improve early identification of PVT in patients with EGVB.

Subgroup analysis of viral etiology (HBV‐ vs. HCV‐related cirrhosis) suggested a potential association with PVT; however, this finding should be interpreted cautiously due to residual confounding, including differences in antiviral treatment exposure, fibrosis stage, and disease duration. Further studies with standardized virological and histological assessment are required to clarify this relationship.

The observed associations between prior endoscopic therapy or splenectomy and PVT are likely influenced in part by confounding by indication. Patients undergoing these interventions generally present with more advanced portal hypertension, and altered splanchnic hemodynamics, which themselves predispose to thrombosis. Although multivariable adjustment was performed, residual confounding from portal flow velocity, spleen size, and variceal severity may persist. Therefore, these associations should be interpreted as risk signals rather than as proof of procedural causality. Prospective designs with detailed hemodynamic profiling are required to further elucidate causality.

This study has several limitations. First, its single‐center retrospective design may limit the generalizability of the findings, and exclusion of patients with incomplete clinical data may introduce selection bias. Future research should adopt a multicenter, prospective design to validate and expand upon the findings of this study. Second, although multivariable analysis was performed, certain potentially important confounders, such as the use of anticoagulants, nonselective *β*‐blockers, and prophylactic anticoagulation strategies after splenectomy, were not systematically captured or adjusted for due to incomplete documentation in the retrospective dataset. Third, the predictive performance of the developed nomogram was moderate, and it has not yet been validated in an independent external cohort; therefore, its robustness and generalizability require further confirmation in future studies.

Despite these limitations, our findings highlight the importance of careful surveillance for PVT in patients with cirrhotic EGVB, particularly in those with a history of splenectomy or endoscopic treatment and elevated DD and FDP levels. Early identification of these high‐risk individuals may facilitate timely intervention and improve clinical outcomes. The proposed nomogram may provide a simple and practical tool for individualized risk stratification in routine clinical practice.

## 5. Conclusion

This study reports a relatively high prevalence of concurrent PVT among patients with cirrhotic EGVB. Splenectomy, prior endoscopic treatment, and elevated DD and FDP levels were associated with the presence of PVT. A predictive nomogram was developed to assist in risk stratification. However, its performance is moderate, and further external validation is required before clinical application. Future multicenter prospective studies are needed to further validate the findings and improve the generalizability of the nomogram.

## Author Contributions

Jiayi Zhang and Yingying He contributed equally to this article.

Jiayi Zhang and Derun Kong conceived and designed the study. Jiayi Zhang and Yingying He contributed to the data acquisition. Jiayi Zhang and Shanshan Wu contributed to the data analysis and interpretation.

## Funding

This study was supported by the Sixth Batch of health and appropriate technology promotion projects in Anhui Province, new technology of comprehensive diagnosis and treatment of esophagogastric varices under endoscopy (SYJS202103); Postgraduate Innovation Research and Practice Program of Anhui Medical University(YJS20230132); and the Department of Gastroenterology, the First Affiliated Hospital of Anhui Medical University, the Key Laboratory of Digestive Diseases of Anhui Province, Hefei 230022, Anhui, China.

## Conflicts of Interest

The authors declare no conflicts of interest.

## Data Availability

The data used to support the findings of the study are available from the corresponding author on reasonable request.
